# Ability of the coronary angiography-derived index of microcirculatory resistance to predict microvascular obstruction in patients with ST-segment elevation

**DOI:** 10.3389/fcvm.2024.1187599

**Published:** 2024-04-22

**Authors:** Xinyan Wen, Zhi Wang, Bo Zheng, Yanjun Gong, Yong Huo

**Affiliations:** Department of Cardiology, Peking University First Hospital, Beijing, China

**Keywords:** coronary angiography-derived index of microvascular resistance, microvascular obstruction, coronary microvascular dysfunction, ST-segment elevation myocardial infarction, predictive ability

## Abstract

**Background:**

The coronary angiography-derived index of microvascular resistance (caIMR) correlates well with the index of microcirculatory resistance (IMR), which predicts microvascular obstruction (MVO). However, the relationship between caIMR and MVO remains unclear.

**Aim:**

To evaluate the predictive ability of caIMR of MVO after ST-segment elevation myocardial infarction (STEMI).

**Methods:**

CaIMR was calculated using computational flow and pressure simulation in patients with STEMI in whom MVO status had been assessed by cardiac magnetic resonance (CMR) after successful primary percutaneous intervention at Peking University First Hospital between December 2016 and August 2019. The clinical, biochemical, echocardiographic, and CMR characteristics were assessed according to MVO status. The predictive value of the clinical parameters and caIMR was evaluated.

**Results:**

Fifty-three eligible patients were divided into an MVO group (*n* = 32) and a no-MVO group (*n* = 21). The caIMR tended to be higher in the MVO group (41.6 U vs. 30.1 U; *p* = 0.136). CaIMR and peak cardiac troponin-I (cTNI) were independent predictors of MVO (per 1-U increment in caIMR: odds ratio [OR] 1.044, 95% confidence interval [CI] 1.004–1.086, *p* = 0.030; per 1 ng/L increase in peak cTNI: OR 1.018, 95% CI 1.003–1.033, *p* = 0.022). In receiver-operating characteristic curve analysis, when a cut-off value of 45.17 U was used, caIMR had some ability to predict MVO (area under the curve 0.622, 95% CI 0.478–0.752, *p* = 0.127).

**Conclusions:**

CaIMR and peak cTNI were independent predictors of short-term MVO in patients with STEMI who had undergone successful primary percutaneous coronary intervention and may help to identify those at high risk of MVO.

## 1 Introduction

The coronary microcirculation consists of pre-arterioles and arterioles that are responsible for maintenance of appropriate myocardial perfusion. Functional and structural abnormalities of the microcirculation contribute to an imbalance in the supply and demand for oxygen, resulting in myocardial ischemia, a condition now referred to as coronary microvascular dysfunction (CMD) (1). CMD has been observed in almost half of patients with ST-segment elevation myocardial infarction (STEMI) despite successful restoration of epicardial coronary artery blood flow (2). However, the coronary microcirculation cannot be visualized by current angiographic systems. Further development of techniques would allow assessment of the functional status of the coronary microcirculation. Measurement of the index of microvascular resistance (IMR) is a gold standard method for functional assessment of the coronary microvasculature (3) and has been recommended for patients with angina who are suspected to have CMD. The IMR is a quantitative and reproducible hyperemic index that is derived from measurements obtained by a pressure-temperature sensor-tipped coronary guidewire. This index is relatively specific for the microcirculation and is not affected by hemodynamic perturbations (4). However, it has had limited application in clinical practice because of the requirement for dedicated intracoronary instruments, need for administration of hyperemic agents, and a relatively long procedural time.

MVO indicates a severe perfusion defect and profound damage to the microcirculation and has a significant association with adverse outcomes, including an increased risk of all-cause mortality and re-admission for heart failure (5). Cardiac magnetic resonance (CMR) is the gold standard for evaluation of MVO but has limited applications in clinical practice because of contraindications to CMR and inaccessibility at the time of primary percutaneous coronary intervention (PPCI).

With advances in technique, caIMR allows the function of the coronary microcirculation to be assessed at the time of the index procedure without the need for a pressure sensor wire and hyperemic agents. Previous studies have confirmed a good correlation and high agreement between caIMR and pressure-wire-based IMR (6–8). Another recent study demonstrated that caIMR has prognostic value in patients with successfully revascularized STEMI (5). However, there is limited information on the relationship between caIMR and MVO. In this study, we evaluated the ability of caIMR to predict the presence of MVO.

## 2 Methods

### 2.1 Study population

Eighty-six patients who were admitted with STEMI to Peking University First Hospital and underwent PPCI with measurement of CMR between December 2016 and August 2019 were enrolled in a prospective cohort study. The cohort study protocol was approved by the Ethics Committee of Peking University First Hospital and all the patients provided written form consent. This is the post-hoc analysis of this cohort study using anonymous clinical data. STEMI was diagnosed based on chest pain lasting for at least 30 min and ST-segment elevation >2 mm in at least two contiguous leads. Data for patients with angiographic images that were inadequate for functional analysis (*n* = 3), those in whom CMR was performed more than 30 days after the intervention (*n* = 19), and those in whom CMR was performed after selective percutaneous intervention (*n* = 11) were excluded. Finally, data for 53 patients with successfully revascularized STEMI were eligible for inclusion in the analysis.

### 2.2 CMR analysis

CMR imaging was performed before discharge (generally 1 week after the index event). All patients were examined using a 1.5-Tesla magnetic resonance imaging scanner (GE Healthcare, Chicago, IL, USA). CMR images were reviewed using commercially available software (Circle Cardiovascular Imaging, Calgary, AB, Canada) by two observers working independently and blinded to the clinical data and the results of physiological assessment. MVO was defined as a hypointense region within a hyperenhanced area on the delayed enhancement images. Hyperenhancement was defined as a region with a signal intensity threshold that was 5 standard deviations above the mean signal intensity of the remote reference myocardium.

### 2.3 Measurement of CaIMR

Coronary angiograms were centrally analyzed in a blinded fashion by a core laboratory using the FlashAngio system (RainMed Medical Technology Co. Ltd., Suzhou, China). The caIMR was estimated in three steps. First, we chose angiograms from at least two different projections to construct three-dimensional mesh models of the coronary arteries. Second, we estimated caFFR using a computational fluid dynamics method. Third, we estimated caIMR using the following formula: Ai et al (9) defined caIMR to assess microcirculation as:

(1) caIMR=L×HMR

*L* represents the length of the target vessel from the inlet to the distal position (*L* = 75 mm). Meuwissen et al. (10) proposed the HMR(hyperemic microvascular resistance) to evaluate microvascular dysfunction as:

(2) $$HMR={\left(Pd\right)}_{\mathrm{hyp}}/V_{\mathrm{hyp}}$$

(3) $$V_{\mathrm{hyp}}=K\times V_{\mathrm{diastole}}$$

$(Pd{)}_{\mathrm{hyp}}$ is the mean pressure at the distal position at maximal hyperemia. And $V_{\mathrm{hyp}}$ is the mean flow velocity at the distal position when hyperemia is maximal $V_{\mathrm{diastole}}$ is the mean flow velocity at the distal position at diastole $V_{\mathrm{diastole}}$ was derived using the TIMI (Thrombolysis in Myocardial Infarction) frame count method. The entire diastolic period provides higher flow velocity and lower microvascular resistance than the whole car diac cycle. And an intracoronary injection of contrast medium can induce some degree of hyperemia. So $V_{\mathrm{hyp}}$ is assumed to be proportional to $V_{\mathrm{diastole}}$. *K* is a constant (*K* = 1.1), obtained from a previous study (6). caFFR is the coronary angiography-derived fractional flow reserve.

(4) $$(Pd{)}_{\mathrm{hyp}}=(Pa{)}_{\mathrm{hyp}}\times caFFR$$

(5) $$caFFR=\left({\left(Pa\right)}_{\mathrm{hyp}}-\Delta P\right)/{\left(Pa\right)}_{\mathrm{hyp}}$$

(6) $$\Delta P=\sum \Delta P_{s}$$

The mean pressure at the aorta at maximal hyperemia $\left({\left(Pa\right)}_{\mathrm{hyp}}\right)$ was estimated on the basis of mean arterial pressure (MAP) during the index procedure, which equals to MAP-MAP*0.2 when MAP ≥ 95 mmHg and MAP-MAP*0.15 when MAP < 95 mmHg, according to a previous study. The pressure drop (Δ*P*_*s*_) across a stenosis was computed from the CFD simulation,which will not be published in details because it is the property of Rainmed Ltd. Δ*P* is the pressure drop along the meshed coronary arteries in the vessel path from the inlet to the most distal position.

According to formulations (Equations 1–4), we can deduce:

(7) $$caIMR=(Pd{)}_{\mathrm{hyp}}\times L/V_{\mathrm{hyp}}={\left(Pa\right)}_{\mathrm{hyp}}\times caFFR\times L/\left(K\times V_{\mathrm{diastole}}\right)$$

Evaluation of caIMR and derivation of the formula have been described in detail by Ai et al. (9). It takes less than 1 min to measure CaIMR.We chose the major epicardial artery with the highest caIMR.

### 2.4 Collection of data

Demographic and clinical data, information on cardiac risk factors, echocardiographic and CMR data, and the results of laboratory investigations during hospitalization were retrieved manually from the hospital electronic medical records system.

### 2.5 Statistical analysis

Categorical variables are expressed as the number and relative frequency (percentage). Continuous variables are shown as the mean ± standard deviation if they were distributed normally and as the median [interquartile range (IQR)] if not. Continuous data with a normal distribution were compared using the Kolmogorov-Smirnov test and by the independent t-test or Mann–Whitney *U* test if not normally distributed. Categorical variables were examined using the chi-squared test. The ability of peak cardiac troponin-I (cTNI) and caIMR to predict the presence of MVO was examined by receiver-operating characteristic curve analysis. The optimal cut-off values were those that yielded the greatest product of sensitivity and specificity in predicting MVO. The areas under the curve (AUCs) for peak cTNI, caIMR, and a combined model were compared using Delong’s test with MedCalc statistical software (MedCalc Software Ltd., Ostend, Belgium). Predictors of MVO were sought in a univariate logistic regression model. All variables with a *p*-value of <0.20 were entered into a multivariable logistic regression model. The results of the logistic regression analysis are presented as odds ratios with 95% confidence intervals (CIs). All other statistical analyses were performed using SPSS statistics version 26.0.0.0 (IBM Corp., Armonk, NY, USA). All tests were two-tailed, and a *p*-value < 0.05 was considered statistically significant.

## 3 Results

**Figure 1:**
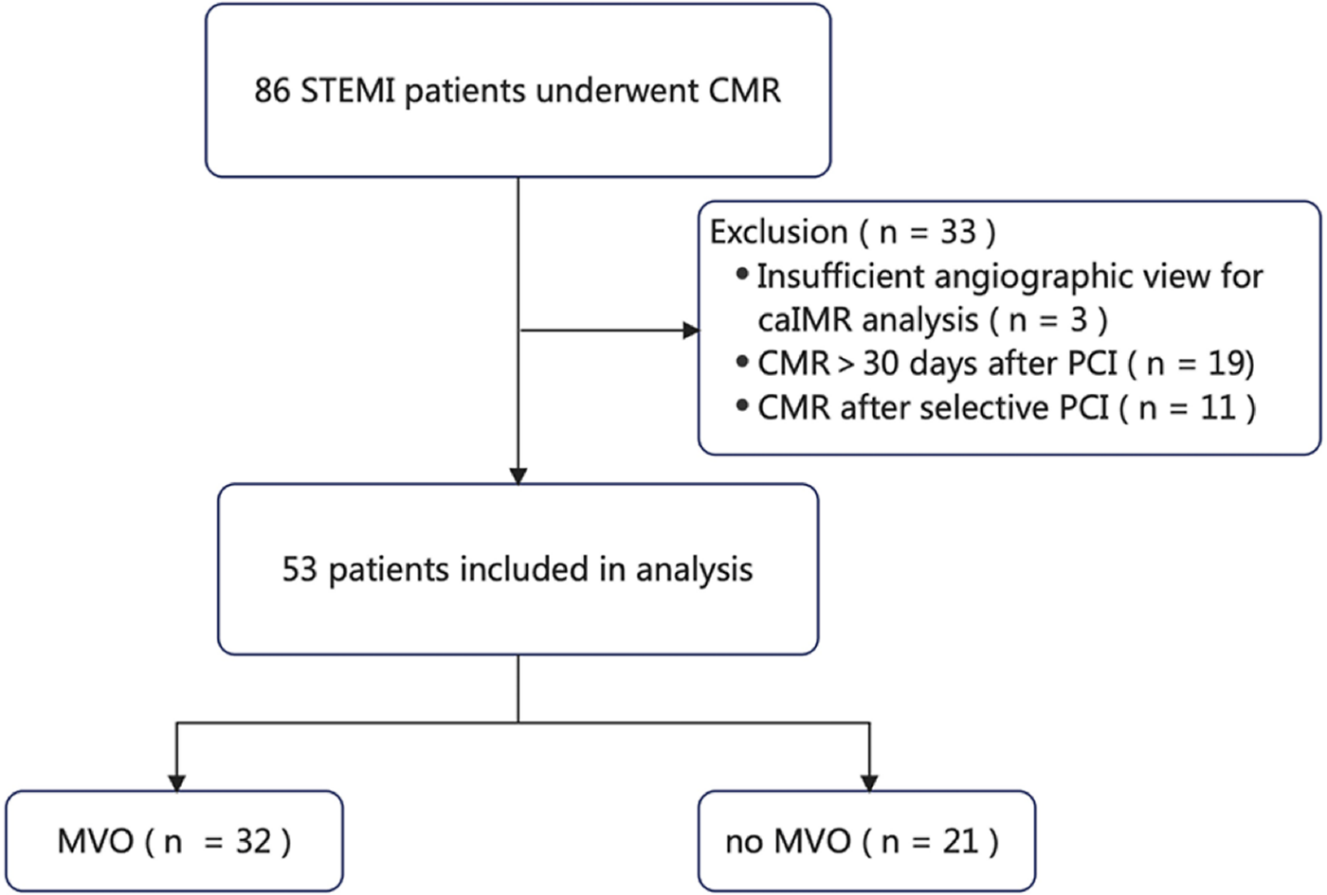
Flow chart showing the procedure used for patient selection. caIMR, coronary angiography-derived index of microvascular resistance; CMR, cardiac magnetic resonance; MVO, microvascular obstruction; PCI, percutaneous coronary intervention; STEMI, ST-segment elevation myocardial infarction.

Fifty-three of the 86 patients with STEMI during our study period met the study inclusion criteria (Figure 1) and were classified based on the presence of MVO into an MVO group (*n* = 32, 60.4%) and a no-MVO group (*n* = 21, 39.6%).

### 3.1 Baseline characteristics

**Figure 2:**
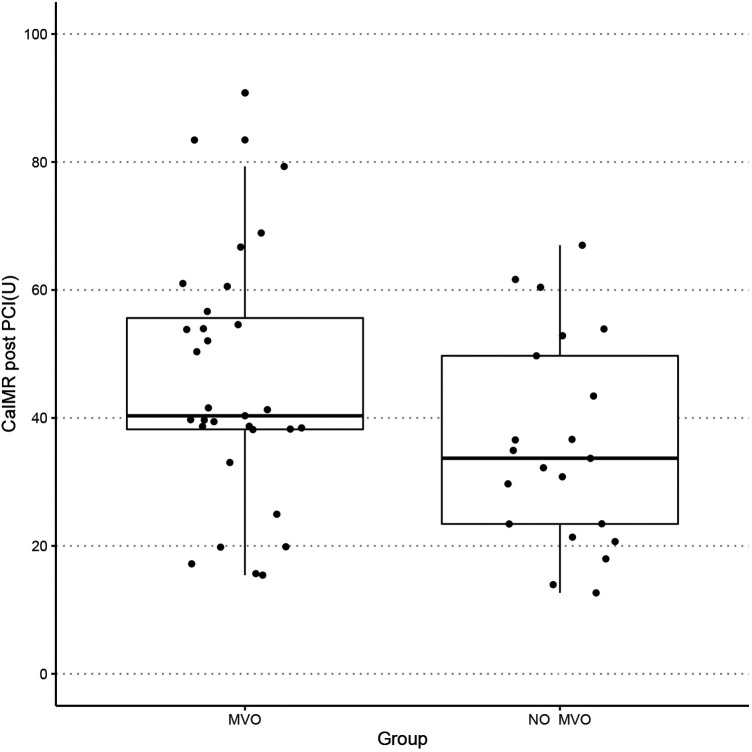
Boxplot showing the distribution of caIMR of the two groups. caIMR, coronary angiography-derived index of microvascular resistance; MVO, microvascular obstruction.

**Figure 3:**
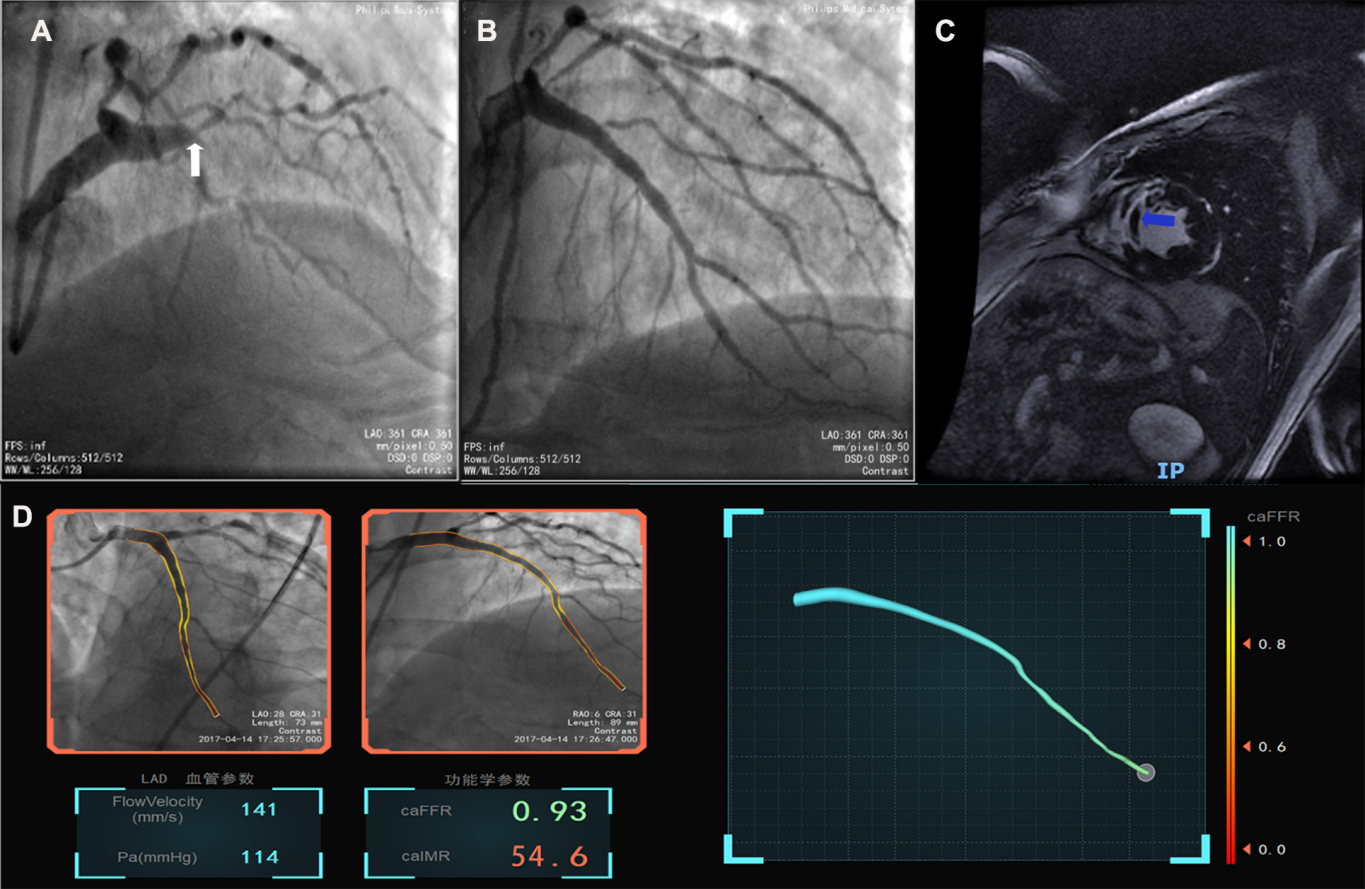
Case example of the coronary angiography-derived index of microcirculatory resistance. (**A**) Pre-surgery angiography; (**B**) post-surgery after revascularization; (**C**), MVO after successful percutaneous intervention in CMR; (**D**), the patient's post-PCI caFFR was normal and caIMR was high (caFFR = 0.93, caIMR = 54.6).

The baseline demographic, clinical, biochemical, and procedural characteristics are presented for the whole cohort and when stratified according to the presence of MVO in Table 1. There was no significant difference in age or sex distribution, cardiac risk factors, or procedural characteristics according to MVO status. Mean door-to-balloon time was 72.5 ± 32.9 min. The most frequent culprit vessel was the left anterior descending artery (60.6%) in the MVO group and the right coronary artery (47.6%) in the no-MVO group. TIMI flow grade 3 was achieved after PCI in 96.2% of cases (MVO group, 93.8%; no-MVO group, 100%). There was no significant between-group difference in low-density lipoprotein cholesterol, high-sensitivity C-reactive protein, estimated glomerular filtration rate, brain natriuretic peptide, or glycated hemoglobin. Patients with MVO had significantly higher values for peak cTnI (110.3 ng/L vs. 30.5 ng/L, *p* = 0.0001) and creatine kinase-myocardial band (273.1 U/L vs. 115.7 U/L, *p* = 0.004) and tended to have a higher caIMR (41.6 U vs. 30.1 U, *p* = 0.136, Figure 2). Figure 3 shows a case examples of patients with microcirculatory dysfunction in the culprit vessel after successful primary PCI.

**Table 1:** Clinical and procedural characteristics.

Clinical data	Total (*n* = 53)	MVO (*n* = 32)	No MVO (*n* = 21)	*P*
Demographics				
Age, y	55.4 ± 9.7	55.5 ± 8.0	55.4 ± 10.8	0.957
Male sex, *n* (%)	50 (94.3%)	29 (90.9%)	21 (100%)	0.403
Body mass index, kg/m^2^	26.0 (22.8, 28.1)	23.6 (21.7, 27.6)	27.0 (25.5, 30.1)	0.013
SBP, mm Hg	134.0 ± 20.5	133.8 ± 18.9	134.5 ± 23.2	0.901
DBP, mm Hg	80.6 ± 13.2	78.6 ± 14.0	83.8 ± 11.6	0.164
Cardiovascular risk factors				
Hypertension, *n* (%)	34 (64.2%)	18 (56.3%)	16 (76.2%)	0.139
Diabetics, *n* (%)	11 (20.8%)	7 (21.9%)	4 (19%)	1.000
CHD family history, *n* (%)	23 (43.4%)	13 (41.9%)	10 (55.6%)	0.357
Smoking, *n* (%)	31 (58.5%)	17 (53.1%)	14 (66.7%)	0.328
Procedural characteristics				
Door-to-balloon time, min	72.5 ± 32.9	68.3 ± 37.2	78.9 ± 24.4	0.253
TIMI grade post PCI				
0	0	0		0.36
1	0	0		
2	2 (3.8%)	2 (6.3%)	0	
3	51 (96.2%)	30 (93.8%)	21 (100%)	
caIMR post PCI, U	38.2 (24.3,54.3)	41.6 (38.2,60.6)	30.1 (21.9,51.3)	0.136
caFFR post PCI	0.9118 ± 0.0500	0.9119 ± 0.0564	0.9115 ± 0.0411	0.976
Culprit artery, LAD %	28 (52.8%)	19 (60.6%)	8 (38.1%)	0.107
Biochemical evaluation				
LDL-C, mg/dl	2.9 (2.4,3.4)	2.8 (2.4, 3.5)	2.8 (2.5, 3.5)	0.880
Hs-CRP, mg/dl	6.2 (2.3,12.1)	11.3 (4.3, 15.1)	2.2 (1.1, 6.3)	0.081
HbA1c,%	5.8 (5.5,6.6)	5.8 (5.7, 7.9)	5.6 (5.5, 6.5)	0.278
eGFR, ml/min/1.73 m^2^	88.3 ± 16.1	89.6 ± 16.6	86.3 ± 15.6	0.473
cTNI peak, ng/L	73.2 (27.4,154.2)	110.3 (73.7,209.8)	30.5 (16.9,55.1)	0.000
CK-MB peak, U/L	238.3 (118.1,371.0)	273.1 (182.5,434.8)	115.7 (78.3,298.5)	0.004
BNP, pg/ml	313.9 ± 309.0	344.2 ± 290.1	267.6 ± 337.8	0.383

Values are expressed as the number (percentage), mean ± standard deviation, or median [interquartile range]. BNP, brain natriuretic peptide; caFFR, angiography-derived fractional flow reserve; caIMR, coronary angiography-derived index of microcirculatory resistance; CK-MB, creatine kinase-myocardial band; CTNI, cardiac troponin I; hs-CRP, high-sensitivity C-reactive protein; LAD, left anterior descending artery; LDL-C, low-density lipoprotein cholesterol; MVO, microvascular obstruction; PCI, percutaneous coronary intervention; TIMI, Thrombolysis in Myocardial Infarction.

### 3.2 Cardiac magnetic resonance and echocardiographic parameters

CMR was performed at a median of 6.87 days after PPCI (IQR 5, 8.5). In total, 94.34% of patients were found to have late gadolinium enhancement on CMR (MVO group, 100%; no-MVO group, 85.7%; *p* = 0.057). Patients in the MVO group had a significantly lower left ventricular ejection fraction (49.8 ± 7.1% vs. 57.9 ± 12.2%, *p* = 0.001), right atrial area (16.0 cm^2^ [95% CI 13.1–17.0] vs. 17.2 cm^2^ [95% CI 14.0–19.7], *p* = 0.024) and a higher end-systolic volume (73.7 ± 19.8 ml vs. 60.8 ± 23.9 ml, *p* = 0.039). There was no significant between-group difference in pulmonary artery systolic pressure, E/A, or E’ (Table 2).

**Table 2:** Echocardiographic and cardiac magnetic resonance parameters.

Variables	Total (*n* = 53)	MVO (*n* = 32)	No MVO (*n* = 21)	p
Echocardiography				
PASP, mm Hg	27.2 (23.8,32.4)	28.8 (26.2,32.3)	23.7 (22.0,33.2)	0.079
E/A	1.085 ± 0.4	1.055 ± 0.4	1.114 ± 0.3	0.564
E’	5.9 ± 1.7	5.9 ± 2.0	5.8 ± 1.3	0.831
IVSD, mm	11.0 (9.9,12.0)	11.0 (9.85,12.0)	11.0 (9.85,12.0)	0.695
LVPWd, mm	11.0 (9.6,11.0)	10.0 (9.7,11.0)	11.0 (9.4,12.0)	0.758
LA, mm	35.3 ± 4.3	35.0 ± 4.6	35.8 ± 4.0	0.518
LVIDs, mm	30.3 ± 5.0	31.2 ± 4.8	28.9 ± 5.0	0.107
LVIDd, mm	47.6 ± 4.6	48.1 ± 4.8	46.8 ± 4.3	0.311
LVEF, %	58.7 ± 9.4	55.5 ± 8.0	63.6 ± 9.3	0.001
CMR				
IVSD, mm	10.9 ± 2.5	10.5 ± 2.5	11.6 ± 2.4	0.129
LVPWDd, mm	6.7 ± 1.9	6.9 ± 2.2	6.4 ± 1.3	0.430
LA area, cm^2^	21.1 ± 4.6	21.2 ± 4.6	20.9 ± 4.6	0.829
RA area, cm^2^	16.0 (13.0,19.0)	16.0 (13.1,17.0)	17.2 (14.0,19.7)	0.024
Edv, ml	144.1 ± 29.7	146.3 ± 30.8	140.5 ± 28.3	0.505
Esv, ml	68.8 ± 22.2	73.7 ± 19.8	60.8 ± 23.9	0.039
LVEF, %	52.9 ± 10.1	49.8 ± 7.1	57.9 ± 12.2	0.001

Values are expressed as the number (percentage), mean ± standard deviation, or median [interquartile range]. CMR, cardiac magnetic resonance; E’, early atrial diastolic annular velocity; Edv, end-diastolic volume; Esv, end-systolic volume; IVSD, interventricular septal thickness at diastole; LVEF, left ventricular ejection fraction; LVPWDd, left ventricular wall thickness at diastole; MVO, microvascular obstruction; PASP, pulmonary artery systolic pressure; RA, right atrium.

### 3.3 Factors that predicted MVO

Multivariable logistic regression analysis identified caIMR to be independently associated with the presence of MVO (per 1 U increase in caIMR: odds ratio 1.044; 95% CI 1.004–1.086, *p* = 0.030). Peak cTNI also independently predicted the presence of MVO (per 1 ng/L increment in peak cTNI: odds ratio 1.018; 95% CI 1.003–1.033, *p* = 0.022; Table 3).

**Table 3:** Independent predictors of microvascular obstruction assessed by cardiac magnetic resonance.

Variable	Univariate analysis		Multivariate analysis	
	OR (95%CI)	*P*	OR (95%CI)	*P*
Age, y	0.998 (0.943, 1.057)	0.956		
Hypertension	2.459 (0.732, 8.457)	0.144	2.134 (0.467, 9.745)	0.328
Diabetes mellitus	0.840 (0.213, 3.321)	0.804		
Current smoker	1.765 (0.563, 5.531)	0.330		
TIMI post PCI ≤ 2	0.873	0.879		
DBP, mm Hg	0.969 (0.928, 1.013)	0.166	0.932 (0.867, 1.003)	0.060
caIMR, U	1.027 (0.996, 1.059)	0.086	1.044 (1.004, 1.086)	0.030
peak cTNI, ng/L	1.014 (1.004, 1.025)	0.007	1.018 (1.003, 1.033)	0.022
peak CK-MB U/L	1.003 (1.000, 1.007)	0.054	0.999 (0.993, 1.004)	0.629
hs-CRP, mg/dl	1.021 (0.972, 1.071)	0.411		

caIMR, coronary angiography-derived index of microvascular resistance; CI, confidence interval; CK-MB, creatine kinase-myocardial band; CMR, cardiac magnetic resonance; cTNI, cardiac troponin I; DBP, diastolic blood pressure; hs-CRP, high-sensitivity C-reactive protein; OR, odds ratio; TIMI, Thrombolysis in Myocardial Infarction.

**Figure 4:**
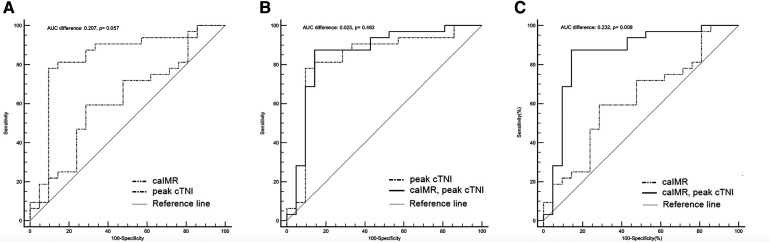
Discriminatory ability for prediction of the presence of MVO. The graphs compare the receiver-operating characteristic curves for the ability of a combination of post-PCI caIMR and peak cTNI (solid black line), peak cTNI (dashed-dotted black line), and post-PCI caIMR (dashed-double-dotted black line) to predict CMR-defined MVO. (**A**) The peak cTNI tended to be a better predictor than caIMR (AUC 0.829 (95% CI 0.700–0.918, *p* < 0.0001 vs. AUC 0.622, 95% CI 0.478–0.752, *p* = 0.127; difference in AUC 0.207, *p* = 0.057). (**B**) There was no significant difference between peak cTNI and the combination of peak cTNI and caIMR (AUC 0.854, 95% CI 0.73–0.936, *p* < 0.001 vs. AUC 0.829, 95% CI 0.700–0.918], *p* < 0.0001; difference in AUC 0.025, *p* = 0.463); (**C**) The AUC was higher for the combination of cTNI and peak cTNI than for caIMR (AUC 0.854, 95% CI 0.73–0.936, *p* < 0.0001 vs. AUC 0.622, 95% CI 0.478–0.752], *p* = 0.127; difference in AUC 0.232, *p* = 0.008). AUC, area under the curve; caIMR, coronary angiography-derived index of microcirculatory resistance; cTNI, cardiac troponin I; CI, confidence interval; MVO, microvascular obstruction.

In ROC curve analysis, the optimal cut-off value for caIMR was 45.17 U (AUC 0.622, 95% CI 0.478–0.752, *p* = 0.127) with a sensitivity of 59.4% and a specificity of 71.4%. Peak cTNI had an AUC of 0.829 (95% CI 0.700–0.918, *p* < 0.0001) for prediction of MVO (optimal cut-off value 71.538 ng/L) and the highest product of sensitivity and specificity (0.781 and 0.905, respectively; Table 4). The predictive value of the peak cTNI tended to be better than that of CaIMR (*p* = 0.0569; Figure 4).

**Table 4:** Performance of independent predictors of microvascular obstruction at optimal cut-off values.

	Optimal value	sensitivity	specificity	PPV	NPV
caIMR, U	45.2	0.594	0.714	0.760	0.536
Peak cTNI, ng/L	71.5	0.781	0.905	0.926	0.731

caIMR, coronary angiography-derived index of microvascular resistance; cTnI, cardiac troponin I; NPV, negative predictive value; PPV, positive predictive value.

Addition of caIMR to peak cTnI did not result in a significantly greater ability to predict MVO than peak cTNI alone (AUC 0.854, 95% CI 0.73–0.936, *p* < 0.001 vs. AUC 0.829, 95% CI 0.700–0.918, *p* < 0.0001; difference in AUC, 0.025, *p* = 0.463). However, the AUC was higher for the combination of peak cTNI and caIMR than for caIMR alone (0.854, 95% CI 0.73–0.936, *p* < 0.0001 vs. 0.622, 95% CI 0.478–0.752, *p* = 0.127; difference in AUC, 0.232, *p* = 0.008).

## 4 Discussion

This study evaluated the ability of caIMR to predict MVO in patients with STEMI after successful PPCI and had two main findings: (1) peak cTNI and caIMR values were independently associated with MVO detected on CMR and (2) a high caIMR tended to be more common in patients with MVO.

The finding of MVO on CMR indicates severe damage at the microvascular level and predicts adverse ventricular remodeling and an increased risk of mortality and morbidity (11–13). However, CMR is not available at the time of percutaneous coronary intervention or widely accessible in routine practice. Therefore, it cannot be used to guide prompt optimized medical therapy and improve the microcirculation. Previous studies of the relationship between IMR and MVO have shown that the IMR is higher in patients with STEMI and MVO (14–16). However, IMR is typically measured using a thermodilution wire with a sensor near its tip, which has less torquability in comparison with workhorse wires, leading to technical difficulty and increased cost (17). Furthermore, IMR requires pharmacological induction of hyperemia, which may be uncomfortable for patients and has a risk of morbidity from arrhythmia. Previous research suggests that caIMR may be a promising alternative indicator of IMR (8, 9). The caIMR is a readily available index and has been confirmed to have an incremental prognostic value over clinical and angiographic factors (6).

In this study, although the between-group difference in caIMR did not reach statistical significance, patients with STEMI and MVO tended to have a higher caIMR than those without MVO. This finding is concordant with data from Cuculi et al., who reported a mean invasive IMR value of 42.9 U in post-STEMI patients with MVO and 31.1 U for those without MVO (14). The failure of our result to reach statistical significance may be attributable to a relatively small infarct size. In our study, the mean left ventricular ejection fraction in patients with MVO was preserved at 49.8%, which indicates a small infarct size with a limited effect on IMR, as demonstrated by Marques et al. (18). According to a previous study, obesity was associated with a high caIMR (19). A relatively higher body mass index of patients in the group without MVO may has played a role in our findings. The limited number of study participants may also have constrained the statistical power of our analysis.

Several previous studies have suggested that cTNI and high-sensitivity cTNI have a predictive value (20–22). Hallen et al. found that the cTNI level at 24 or 48 h was independently associated with MVO (23). Other studies have shown that the peak cTNI concentration correlates well with the extent of MVO and infarct mass and can predict clinical outcomes (24–27). Thus, patients with MVO have a larger myocardial infarct size than those without MVO. In our study, the peak cTNI level was an independent predictor of the presence of MVO, which is consistent with previous reports (23, 28).

CaIMR is a readily available, quantitative, and wire-free method that can be used to assess acute microvascular dysfunction, which has been shown to correlate well with IMR and be an independent predictor of long-term outcomes. In line with another recent study (28), we found caIMR to be an independent predictor of MVO that may be used to identify patients at high risk of developing MVO and guide personalized precision medicine during and following PPCI. Although the ability of caIMR to discriminate MVO was inferior to that of peak cTNI or a combination of peak cTNI and caIMR, caIMR is an index that is readily available in a catheterization laboratory and has advantages over other methods at the time of STEMI. The clinical implication of this finding is that caIMR is useful for risk stratification of persons who may benefit most from early adjunctive treatment and individualized treatment strategies aimed at microvascular recovery following PPCI.

Efforts are ongoing to identify effective adjunctive therapies to improve microvascular function and clinical outcomes after STEMI, including intracoronary administration of glycoprotein IIb/IIIa inhibitors, use of coronary vasodilators, and periprocedural anti-inflammatory interventions (29). CaIMR may help us to detect patients at high risk of developing MVO and adverse clinical outcomes. Perhaps most important, better assessment of microvascular dysfunction may pave the way for development of therapies aimed specifically at microvascular dysfunction.

### 4.1 Limitations

This study has several limitations, First, it had a single-center observational design and included a small sample size. Therefore, it may not have been fully representative of patients encountered in routine clinical practice. Second, we did not evaluate the extent of the microvasculature or quantify the area at risk or infarct size using CMR, which limits any further exploration of the association between caIMR and MVO. Third, we did not measure the IMR derived using a conventional pressure wire. Therefore, we could not validate the diagnostic accuracy of caIMR. However, a recent study that used the same software and methodology as in our study to calculate caIMR confirmed a high correlation between caIMR and IMR (9). Fourth, clinical outcomes are not included in this study. Fifth, caIMR was measured based on the angiography-derived mean pressure and diastolic flow velocity in the contrast-induced sub-hyperemia which may result the discordance between caIMR and IMR. However, previous study has confirmed the good diagnostic of caIMR in patients with stable/unstable angina pectoris (9). Sixth, caIMR is not repeatable. CFR (coronary flow reserve) by transthoracic doppler methods is a noinvasive method with high feasibility, which has been proved to have excellent prediction of clinical outcomes, precise discrimination between microcircle and epicardial conduit status and excellent assessment of the severity of coronary artery stenosis (30–32). CFR may be integrated with caIMR during the post angioplasty follow-up in our future study.

## 5 Conclusion

Peak cTNI and caIMR are predictors of MVO in patients with STEMI that has been successfully revascularized. caIMR holds promise as a method for evaluation of CMD and prediction of the presence of MVO at the time of STEMI, which would facilitate prompt adjunctive therapy aimed at microvascular recovery. A further study is warranted to clarify in more detail the diagnostic performance and prognostic value of caIMR in patients with STEMI.

## Data Availability

The raw data supporting the conclusions of this article will be made available by the authors, without undue reservation.

## References

[1] Camici PG d'Amati G Rimoldi O. Coronary microvascular dysfunction: mechanisms and functional assessment. Nat Rev Cardiol. (2015) 12(1):48–62. 10.1038/nrcardio.2014.160 25311229

[2] van Kranenburg M Magro M Thiele H de Waha S Eitel I Cochet A Prognostic value of microvascular obstruction and infarct size, as measured by CMR in stemi patients. JACC Cardiovasc Imaging. (2014) 7(9):930–9. 10.1016/j.jcmg.2014.05.010 25212799

[3] Fearon WF Kobayashi Y. Invasive assessment of the coronary microvasculature: the Index of microcirculatory resistance. Circ Cardiovasc Interv. (2017) 10(12). 10.1161/CIRCINTERVENTIONS.117.005361 29222132

[4] Ng MK Yeung AC Fearon WF. Invasive assessment of the coronary microcirculation: superior reproducibility and less hemodynamic dependence of Index of microcirculatory resistance compared with coronary flow reserve. Circulation. (2006) 113(17):2054–61. 10.1161/CIRCULATIONAHA.105.603522 16636168

[5] Scarsini R Shanmuganathan M De Maria GL Borlotti A Kotronias RA Burrage MK Coronary microvascular dysfunction assessed by pressure wire and CMR after stemi predicts long-term outcomes. JACC Cardiovasc Imaging. (2021) 14(10):1948–59. 10.1016/j.jcmg.2021.02.023 33865789

[6] Choi KH Dai N Li Y Kim J Shin D Lee SH Functional coronary angiography-derived Index of microcirculatory resistance in patients with ST-segment elevation myocardial infarction. JACC Cardiovasc Interv. (2021) 14(15):1670–84. 10.1016/j.jcin.2021.05.027 34353599

[7] De Maria GL Scarsini R Shanmuganathan M Kotronias RA Terentes-Printzios D Borlotti A Angiography-Derived Index of microcirculatory resistance as a novel, pressure-wire-free tool to assess coronary microcirculation in ST elevation myocardial infarction. Int J Cardiovasc Imaging. (2020) 36(8):1395–406. 10.1007/s10554-020-01831-7 32409977 PMC7381481

[8] Mejia-Renteria H Lee JM Choi KH Lee SH Wang L Kakuta T Coronary microcirculation assessment using functional angiography: development of a wire-free method applicable to conventional coronary angiograms. Catheter Cardiovasc Interv. (2021) 98(6):1027–37. 10.1002/ccd.29863 34242489

[9] Ai H Feng Y Gong Y Zheng B Jin Q Zhang HP Coronary angiography-derived index of microvascular resistance. Front Physiol. (2020) 11:605356. 10.3389/fphys.2020.605356 33391020 PMC7772433

[10] Meuwissen M Chamuleau SA Siebes M Schotborgh CE Koch KT de Winter RJ Role of variability in microvascular resistance on fractional flow reserve and coronary blood flow velocity reserve in intermediate coronary lesions. Circulation. (2001) 103(2):184–7. 10.1161/01.cir.103.2.184 11208673

[11] Eitel I Kubusch K Strohm O Desch S Mikami Y de Waha S Prognostic value and determinants of a hypointense infarct core in T2-weighted cardiac magnetic resonance in acute reperfused ST-elevation-myocardial infarction. Circ Cardiovasc Imaging. (2011) 4(4):354–62. 10.1161/CIRCIMAGING.110.960500 21518773

[12] Rochitte CE Lima JA Bluemke DA Reeder SB McVeigh ER Furuta T Magnitude and time course of microvascular obstruction and tissue injury after acute myocardial infarction. Circulation. (1998) 98(10):1006–14. 10.1161/01.cir.98.10.1006 9737521

[13] Wu KC Zerhouni EA Judd RM Lugo-Olivieri CH Barouch LA Schulman SP Prognostic significance of microvascular obstruction by magnetic resonance imaging in patients with acute myocardial infarction. Circulation. (1998) 97(8):765–72. 10.1161/01.cir.97.8.765 9498540

[14] Cuculi F De Maria GL Meier P Dall'Armellina E de Caterina AR Channon KM Impact of microvascular obstruction on the assessment of coronary flow reserve, Index of microcirculatory resistance, and fractional flow reserve after st-segment elevation myocardial infarction. J Am Coll Cardiol. (2014) 64(18):1894–904. 10.1016/j.jacc.2014.07.987 25444143

[15] McGeoch R Watkins S Berry C Steedman T Davie A Byrne J The index of microcirculatory resistance measured acutely predicts the extent and severity of myocardial infarction in patients with st-segment elevation myocardial infarction. JACC Cardiovasc Interv. (2010) 3(7):715–22. 10.1016/j.jcin.2010.04.009 20650433

[16] Yoo SH Yoo TK Lim HS Kim MY Koh JH. Index of microcirculatory resistance as predictor for microvascular functional recovery in patients with anterior myocardial infarction. J Korean Med Sci. (2012) 27(9):1044–50. 10.3346/jkms.2012.27.9.1044 22969250 PMC3429821

[17] Cottens D Maeremans J Vrolix M Van Lierde J Dens J Ferdinande B. Ffr pressure wire comparative study: piezoresistive versus optical sensor. Acta Cardiol. (2021) 477:1–6. 10.1080/00015385.2021.1939510 34218723

[18] Marques KM Knaapen P Boellaard R Westerhof N Lammertsma AA Visser CA Hyperaemic microvascular resistance is not increased in viable myocardium after chronic myocardial infarction. Eur Heart J. (2007) 28(19):2320–5. 10.1093/eurheartj/ehm309 17656351

[19] Lee JM Layland J Jung JH Lee HJ Echavarria-Pinto M Watkins S Integrated physiologic assessment of ischemic heart disease in real-world practice using Index of microcirculatory resistance and fractional flow reserve: insights from the international Index of microcirculatory resistance registry. Circ Cardiovasc Interv. (2015) 8(11):e002857. 10.1161/CIRCINTERVENTIONS.115.002857 26499500

[20] Bogaert J Kalantzi M Rademakers FE Dymarkowski S Janssens S. Determinants and impact of microvascular obstruction in successfully reperfused ST-segment elevation myocardial infarction. Assessment by magnetic resonance imaging. Eur Radiol. (2007) 17(10):2572–80. 10.1007/s00330-007-0627-9 17361420

[21] Feistritzer HJ Reinstadler SJ Klug G Reindl M Wöhrer S Brenner C Multimarker approach for the prediction of microvascular obstruction after acute ST-segment elevation myocardial infarction: a prospective, observational study. BMC Cardiovasc Disord. (2016) 16(1):239. 10.1186/s12872-016-0415-z 27894261 PMC5126989

[22] Schaaf M Huet F Akodad M Gorce-Dupuy AM Adda J Macia JC Which high-sensitivity troponin variable best characterizes infarct size and microvascular obstruction? Arch Cardiovasc Dis. (2019) 112(5):334–42. 10.1016/j.acvd.2018.12.001 30777683

[23] Hallen J Jensen JK Buser P Jaffe AS Atar D. Relation of cardiac troponin I and microvascular obstruction following st-elevation myocardial infarction. Acute Card Care. (2011) 13(1):48–51. 10.3109/17482941.2010.538698 21244230

[24] Chin CT Wang TY Li S Wiviott SD deLemos JA Kontos MC Comparison of the prognostic value of peak creatine kinase-mb and troponin levels among patients with acute myocardial infarction: a report from the acute coronary treatment and intervention outcomes network registry-get with the guidelines. Clin Cardiol. (2012) 35(7):424–9. 10.1002/clc.21980 22434769 PMC6652484

[25] Ingkanisorn WP Rhoads KL Aletras AH Kellman P Arai AE. Gadolinium delayed enhancement cardiovascular magnetic resonance correlates with clinical measures of myocardial infarction. J Am Coll Cardiol. (2004) 43(12):2253–9. 10.1016/j.jacc.2004.02.046 15193689

[26] Giannitsis E Steen H Kurz K Ivandic B Simon AC Futterer S Cardiac magnetic resonance imaging study for quantification of infarct size comparing directly serial versus single time-point measurements of cardiac troponin T. J Am Coll Cardiol. (2008) 51(3):307–14. 10.1016/j.jacc.2007.09.041 18206741

[27] Pernet K Ecarnot F Chopard R Seronde MF Plastaras P Schiele F Microvascular obstruction assessed by 3-tesla magnetic resonance imaging in acute myocardial infarction is correlated with plasma troponin I levels. BMC Cardiovasc Disord. (2014) 14:57. 10.1186/1471-2261-14-57 24886208 PMC4013057

[28] Shin D Kim J Choi KH Dai N Li Y Lee SH Functional angiography-derived Index of microcirculatory resistance validated with microvascular obstruction in cardiac magnetic resonance after stemi. Rev Esp Cardiol (Engl Ed). (2022) 75:786–96. 10.1016/j.rec.2022.01.004 35249841

[29] Kleinbongard P Heusch G. A fresh look at coronary microembolization. Nat Rev Cardiol. (2022) 19(4):265–80. 10.1038/s41569-021-00632-2 34785770 PMC8593642

[30] Caiati C Pollice P Iacovelli F Sturda F Lepera ME. Accelerated stenotic flow in the left anterior descending coronary artery explains the causes of impaired coronary flow reserve: an integrated transthoracic enhanced doppler study. Front Cardiovasc Med. (2023) 10:1186983. 10.3389/fcvm.2023.1186983 37745100 PMC10515222

[31] Caiati C Lepera ME Pollice P Iacovelli F Favale S. A new noninvasive method for assessing mild coronary atherosclerosis: transthoracic convergent color doppler after heart rate reduction. Validation vs. Intracoronary ultrasound. Coron Artery Dis. (2020) 31(6):500–11. 10.1097/MCA.0000000000000873 32271240

[32] European Commission. European Commission Referral Guidelines for Imaging (2000). 1–125.

